# Increased trefoil factor 2 levels in patients with chronic kidney disease

**DOI:** 10.1371/journal.pone.0174551

**Published:** 2017-03-29

**Authors:** Diana Lebherz-Eichinger, Bianca Tudor, Hendrik J. Ankersmit, Thomas Reiter, Martin Haas, Elisa Einwallner, Franziska Roth-Walter, Claus G. Krenn, Georg A. Roth

**Affiliations:** 1 Department of Anesthesiology, General Intensive Care and Pain Medicine, Medical University of Vienna, Vienna, Austria; 2 Christian Doppler Laboratory for Cardiac and Thoracic Diagnosis and Regeneration, Medical University of Vienna, Vienna, Austria; 3 RAIC Laboratory 13C1, Medical University of Vienna, Vienna, Austria; 4 Department of Thoracic Surgery, Medical University of Vienna, Vienna, Austria; 5 Division of Nephrology and Dialysis, Department of Medicine III, Medical University of Vienna, Vienna, Austria; 6 Department of Cardiology, University Hospital St. Pölten, St. Pölten, Austria; 7 Department of Laboratory Medicine, Medical University of Vienna, Vienna, Austria; 8 Comparative Medicine, Messerli Research Institute, University of Veterinary Medicine of Vienna, Medical University of Vienna and University of Vienna, Vienna, Austria; The University of Tokyo, JAPAN

## Abstract

In chronically damaged tissue, trefoil factor family (TFF) peptides ensure epithelial protection and restitution. In chronic kidney disease (CKD), TFF1 and TFF2 are reported to be upregulated. Especially in the early phase, CKD is associated with silently ongoing renal damage and inflammation. Moreover, many patients are diagnosed late during disease progression. We therefore sought to investigate the potential of TFF2 as biomarker for CKD. We followed 118 patients suffering from predialysis CKD and 23 healthy volunteers. TFF2 concentrations were measured using ELISA. Our results showed, that median TFF2 serum levels were significantly higher in patients with later CKD stages as compared to healthy controls (p < 0.001) or early stages (p < 0.001). In patients with mid CKD stages TFF2 serum levels were significantly higher than in healthy controls (p = 0.002). Patients with early or mid CKD stages had significantly higher TFF2 urine concentrations than later CKD stages (p < 0.001 and p = 0.009, respectively). Fractional TFF2 excretion differed significantly between early CKD stages and healthy controls (p = 0.01). ROC curve showed that TFF2 levels can predict different CKD stages (AUC > 0.75). In conclusion, urine and serum TFF2 levels of CKD patients show a different profile dependent on CKD stages. Whereas TFF2 urine levels continuously decreased with disease progression, TFF2 serum concentrations progressively increased from the early to later CKD stages, indicating changes in renal function and offering the potential to examine the course of CKD.

## Introduction

Chronic kidney disease (CKD) describes the steady loss of kidney function and is defined by kidney damage or by an estimated glomerular filtration rate (eGFR) of less than 60 mL per minute per 1.73 m^2^ body-surface for a minimum of three months [1]. CKD patients are at increased risk of developing serious complications like cardiovascular diseases and even a slight decrease in GFR can result in anaemia or bone disease [1–3]. The progression of CKD proceeds silently, resulting in patients diagnosed at a state where most therapeutic options to prevent adverse outcomes are insufficient [4]. Consequently, the early detection of individuals at risk is highly desirable to initiate a timely treatment to prevent disease progression.

The decline of kidney function is associated with ongoig inflammatory response and increased cell death [5–7]. Trefoil factor (TFF) peptides have been shown to be upregulated in the damaged kidney, obviously to ameliorate epithelial destruction [8–10]. These proteins are secreted by several mucine-producing epithelial cells and are involved in mucousal healing. TFF peptides facilitate epithelial restitution and regeneration processes by the induction of cell migration, angiogenesis, and by raising cell resistance to proapoptotic stimuli [11–13]. Restitution describes the recovery of mucosal continuity via elongation and cell migration to cover damaged denuded areas.

TFF peptides are evolutionally a highly conserved group of proteins and are named after the so called trefoil domain, which consists of a three-looped structure of cysteine residues. The protein family comprises three members: TFF1, TFF2, and TFF3, with TFF2 to be discovered first of all [11]. TFF peptides are secreted by most epithelial tissues that contain mucus-secreting cells including renal tubular epithelial cells in the kidney. In animal models for kidney toxicity, TFF3 is already an accepted biomarker for the estimation of renal damage [14]. Increased levels of TFF3 and TFF1 have also been detected in patients with CKD and closely correlated with renal function [9,10,15].

To evaluate if TFF2 levels also change during progression of CKD we investigated TFF2 levels in serum and urine of 118 patients suffering from early, mid or later stages of CKD in comparison to healthy controls. Furthermore, we analysed the potential of TFF2 as biomarker for renal damage and calculated fractional TFF2 excretion to unravel changes in renal excretion irrespective of the glomerular filtration rate.

## Methods

### Patients

This study has been approved by the ethics committee of the Medical University of Vienna and was conducted according to the Helsinki Declaration of 1975.

CKD patients were diagnosed, screened and followed up at the Division of Nephrology and Dialysis, Department of Medicine III in the Medical University of Vienna. CKD was defined as decreased glomerular filtration rate and/or the presence of kidney damage in accordance with the K/DOQI criteria [1]. For calculating eGFR the formular described in the 2002 guidelines was used to allow comparisons to already published studies dealing about TFF levels in CKD. 118 patients with CKD stage 1 to 5 were included into the study during a follow-up appointment at the outpatient clinic of the Division of Nephrology and Dialysis. 23 healthy volunteers served as controls. In the control group, abdominal pain over the last four weeks, kidney diseases, and pregnancy were excluded via anamnesis. All participiants signed an informed consent before inclusion into the study.

Table 1 shows the patients’ diagnoses, baseline demographics and laboratory values.

**Table 1:** Patients baseline demographic and laboratory data. Underlying kidney diseases.

	All patients	Early CKD stages (stage 1, stage 2)	Mid CKD stages(stage 3)	Later CKD stages(stage 4, stage 5)	Controls
N	118	33 (28%)	39 (33%)	46 (39%)	23
Age (years)	56 (19–88)	44 (19–80)	61 (23–78)	61 (20–88)	39 (21–67)
Male/Female (%)	57/43	48/52	69/31	52/48	65/35
Weight (kg)	75 (36–120)	80 (54–120)	77.5 (50–118)	72.5 (36–116)	
Height (cm)	172 (120–198)	172 (152–198)	174 (147–185)	166 (120–190)	
BMI	25.3 (15.4–39)	26 (21.8–39)	25.6 (18.3–33.3)	25.7 (15.4–36)	
Smoker/Non-smoker (%)	60/40	67/33	65/35	54/46	
Kidney disease					
Glomerulonephritis	34	12	9	13	
Vascular nephropathy	20	3	8	9	
Diabetic nephropathy	12	2	7	3	
Polycystic kidney disease	10	3	2	5	
Hereditary angiomyolipoma	1	1	-	-	
Interstitial nephropathy	7	4	1	2	
Urine stasis	6	1	1	4	
Nephrectomy	4	1	-	3	
Urothelial/Renal cell carcinoma	3	1	2	-	
Unknown	21	5	9	7	
Creatinine (mg dL^-1^)	1.86(0.72–6.88)	0.98(0.72–1.52)	1.68(1.02–2.34)	3.54(3.14–6.88)	0.99(0.77–1.3)
Urea(mg dL^-1^)	31.9(7.1–91.2)	12.9(7.1–33.4)	30.5(11.6–64.1)	55.6(23.8–91.2)	13.1(0–20)
Urine creatinine (mg dL^-1^)	70.9(12.7–294.5)	72.4(69.9–252.9)	81.1(12.7–294.5)	56.6(17.7–172.1)	138.5(31.4–418.6)
Urine urea Nitrogen(mg dL^-1^)	844(247–2557)	976(247–2557)	939(291–2464)	676(267–1381)	
Urine protein (g L^-1^)	0.47(< 0.05–6.94)	0.21(< 0.05–4.45)	0.47(< 0.05–2.79)	0.74(< 0.05–6.94)	
Protein:creatinine ratio (g gCrea^-1^)	0.29 (0–8.99)	0.07 (0–4.47)	0.13 (0–7.72)	1.09 (0–8.99)	
CRP (mg dL^-1^)	0.28(0.02–8.53)	0.15(0.02–4.31)	0.5(0.03–3.87)	0.3(0.04–8.53)	

Patients with urothelial or renal cell carcinoma underwent chemotherapy. Drug abuse could be verified in two patients diagnosed with mid CKD and in one patient with ESRD. Data are given as median with range. CKD, chronic kidney disease.

### Laboratory data

Blood and spontaneous urine samples were obtained from all patients and healthy volunteers. Additionally, 24-hour urine samples were obtained in a subgroup of 34 patients. Serum samples were collected in a 9 mL Z Serum Clot Activator Tube and the urine in Vacuette^®^ Urine Tubes (both provided by Greiner Bio-One International GmbH, Austria). Serum and urine samples were centrifuged at 2000 RCF for 10 min at 4°C. Aliquots were then transferred in tubes, snap frozen and stored at—80°C.

TFF2 concentrations were measured by an enzyme-linked immunosorbent assay (ELISA) kit (Human TFF2 DuoSet, R&D Systems, Minneapolis, Minnesota), based on Vestergaard *et al* [16]. The measurement was performed according to the manufacturer’s instructions. Serum samples were diluted 1:5 and urine samples 1:125 to obtain protein concentrations within the standard curve. Tetramethylbenzidin (TMB; Sigma, St. Louis, Missouri) was used as substrate and color reaction was stopped by adding 1 N sulfuric acid solution (in aqua bidistilled diluted 95–98% sulfuric acid, Merck KGaA, Darmstadt, Germany). The optical density was immediately measured with a Victor 3 microplate reader at a 450 nm wavelength. To overcome potential methodological deviations, samples were randomized before analysis. Spike/recovery and linearity testing was performed in accordance with the spike, recovery, and linearity protocol for validating untested samples of R&D systems. Therefore, known concentrations of TFF2 were admixed to urine and serum samples and the used buffer. Additionally, serial dilution of spiked and unspiked samples were performed for linearity testing. By the evaluation of pure and diluted samples (spiked and unspiked) the recovery was obtained. The average recovery was 94% and the average linearity was 105%. Multiple testing on different plates revealed a coefficient of variation of 3% for intra-assay precision and 9% for inter-assay precision. The kit’s sensitivity was obtained by the summation of two standard deviations to the mean optical density of twenty-two zero samples and by the following calculation of the corresponding concentration. The minimum detectable concentration was 23 pg/mL. No cross-reactivity to TFF1 or TFF3 could be detected by testing the kit’s specificity.

The fractional TFF2 peptide excretion was calculated using the formula: ((urinary TFF2 peptide x serum creatinine) / (serum TFF2 peptide x urinary creatinine)) x 100.

### Statistical analysis

TFF2 concentrations in serum and urine and fractional TFF2 excretion were analysed in all groups (early, mid, and later CKD stages as well as in the control group). Gaussian distribution was assessed with the D’Agostino-Pearson normality test. Normal distribution could not be verified for all analyzed groups. Consequently, the non-parametric Mann-Whitney-test (two-tailed) was used to compare TFF2 levels. TFF2 levels within CKD entities diagnosed in more then 10 patients (glomerulonephritis, vascular nephropathy, diabetic nephropathy) were analysed with the Mann-Whitney-test (two-tailed). After Bonferroni correction for multiple comparisons, an individual p < 0.016 was necessary to achieve statistical significance at the 5% level.

The correlation analysis between the TFF2 serum and urine concentrations and several clinical parameters (age, serum creatinine, urinary protein, creatinine clearance, urinary protein:creatinine ratio, eGFR, CRP) were performed by Spearman's correlation coefficient. Unless otherwise specified, data are given as median and range. All statistical analysis was performed with the GraphPad Prism Version 5.01 (GraphPad Software, Inc. California, US).

## Results

### Absolute and fractional TFF2 levels in CKD stages

**Fig 1:**
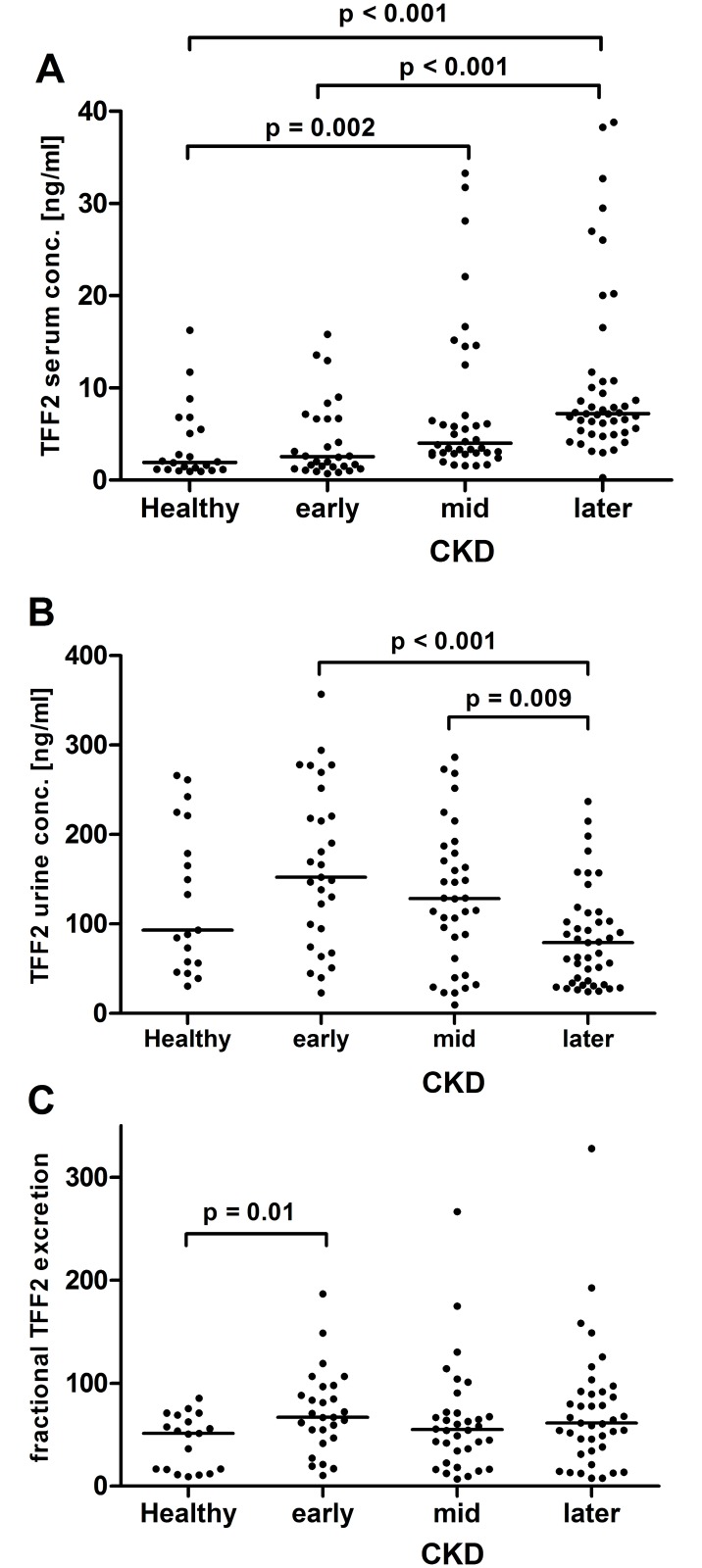
TFF2 levels. Panel A: TFF2 serum levels, one data point outside the axis limits in early CKD stages. Panel B: TFF2 urine concentrations. Panel C: Fractional TFF2 excretion, one data point outside the axis limits in later CKD stages. Each dot represents an individual patient. The line indicates the median. CKD, chronic kidney disease. Only significant p-values are given.

Total TFF2 serum concentrations were significantly higher in mid and later CKD stages as compared to healthy controls (Fig 1A). Furthermore, TFF2 serum levels in later CKD stages differed significantly from early stages (Fig 1A). Urine TFF2 levels were significantly higher in early and mid CKD stages as compared to later stages (Fig 1B). The fractional TFF2 excretion was higher in early stages as compared to healthy controls, but not in mid and later stages of CKD (Fig 1C)

### Absolute and fractional TFF2 levels in CKD entities

TFF2 serum levels were significantly higher in patients with vascular nephropathy or diabetic nephropathy as compared to patients suffering from glomerulonephritis (Table 2). Fractional TFF2 excretion was significantly elevated in patients with glomerulonephritis as compared to patients with vascular nephropathy (Table 2). No significant differences in TFF2 levels were found between vascular and diabetic nephropathy (Table 2).

**Table 2:** TFF2 serum and urine concentrations as well as fract TFF2 excretion analysed within nephropathies diagnosed in more than 10 patients.

	Glomerulonephritis	Vasc. nephropathy	Diabetic nephropathy
TFF2 serum conc., (ng ml^-1^)	3.6 (1–10)	9.5 (2–33.3)*	13.5 (4.2–28.1)*
TFF2 urine conc., (ng ml^-1^)	114.2 (9.5–272.7)	86.2 (23.3–277.3)	90.4 (29.5–286.6)
fract. TFF2 excretion	67.1 (9.6–328)	16.1 (6.8–77.7)*	38.2 (16.6–130.3)

All patients with the given disease were included, Asterisks indicate significant differences in values between vascular nephropathy or diabetic nephropathy as compared to glomerulonephritis (p < 0.01). No significant differences between vascular nephropathy and diabetic nephropathy or within urine concentrations could be found.

### Correlations of total TFF serum and urine levels with clinical and kidney function parameters

**Fig 2:**
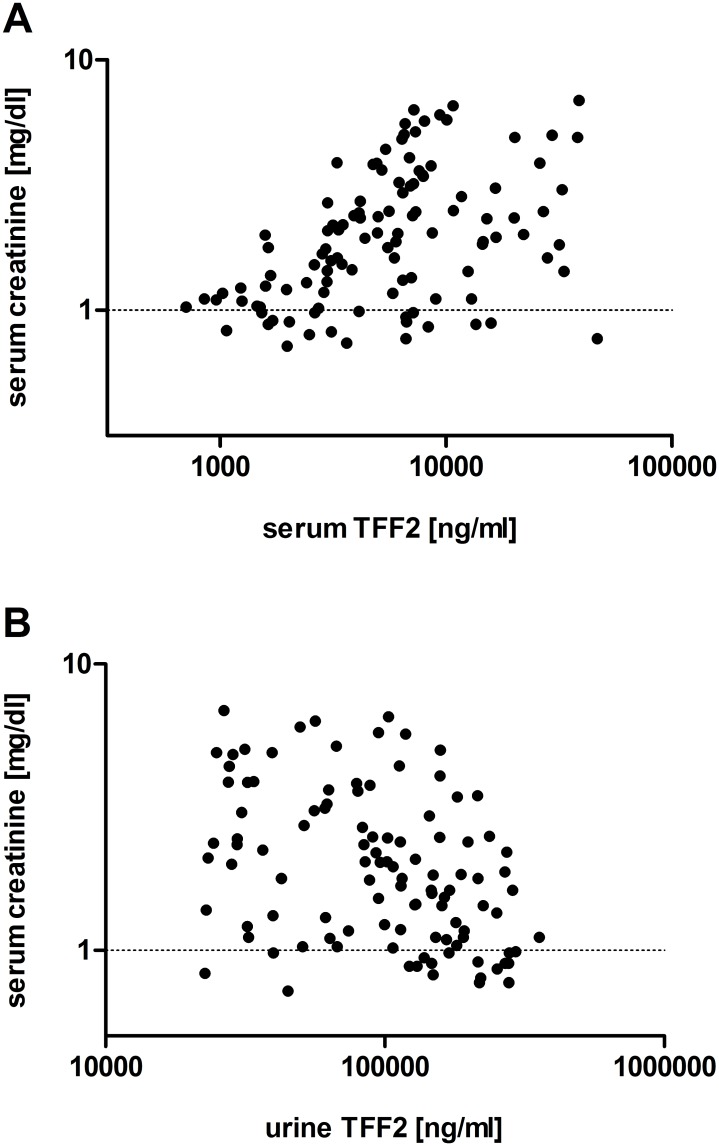
Correlations. Panel A: Serum TFF2 and serum creatinine correlated significantly using Spearman's rank correlation coefficient (Spearman's r = 0.44, p < 0.001, 113 pairs). The X and the Y-axis are given as log scale. Panel B: Urine TFF2 and serum creatinine negatively correlated using Spearman's rank correlation coefficient (Spearman's r = 0.4, p < 0.001, 111 pairs). The X and the Y-axis are given as log scale.

There was a significant negative correlation between TFF2 serum levels and creatinine clearance in the subgroup of patients with a 24-hour urine analysis (Table 3). Age positively correlated with serum TFF2 levels, but the correlation coefficient was only moderate (< 0.4) (Table 3). There was a significant correlation with TFF2 concentrations and eGFR (Table 3). Serum TFF2 positively correlated with serum creatinine, as depicted in Fig 2A. Urine TFF2 concentrations significantly correlated with serum creatinine (Table 3, Fig 2B). All correlations are given in Table 3 and Fig 2.

**Table 3:** Correlation of TFF2 serum and urine concentrations with clinical and kidney function parameters.

	Serum TFF2	Urine TFF2
Age (113 pairs)	r = 0.34, p < 0.001*	r = 0.05, p = 0.6
Urine protein (111 pairs)	r = 0.22, p = 0.02	r = 0.05, p = 0.6
Creatinine clearance (32 pairs)	r = - 0.64, p < 0.001*	r = 0.18, p = 0.3
Protein:creatinine ratio(111 pairs)	r = 0.21, p = 0.03	r = - 0.1, p = 0.3
eGFR (113 pairs)	r = - 0.43, p < 0.001*	r = 0.37, p < 0.001*
CRP (113 pairs)	r = 0.22, p = 0.2	r = - 0.18, p = 0.9

Asterisks indicate significance (* p < 0.001).

### ROC curve analysis of total TFF2 serum and urine levels

**Fig 3:**
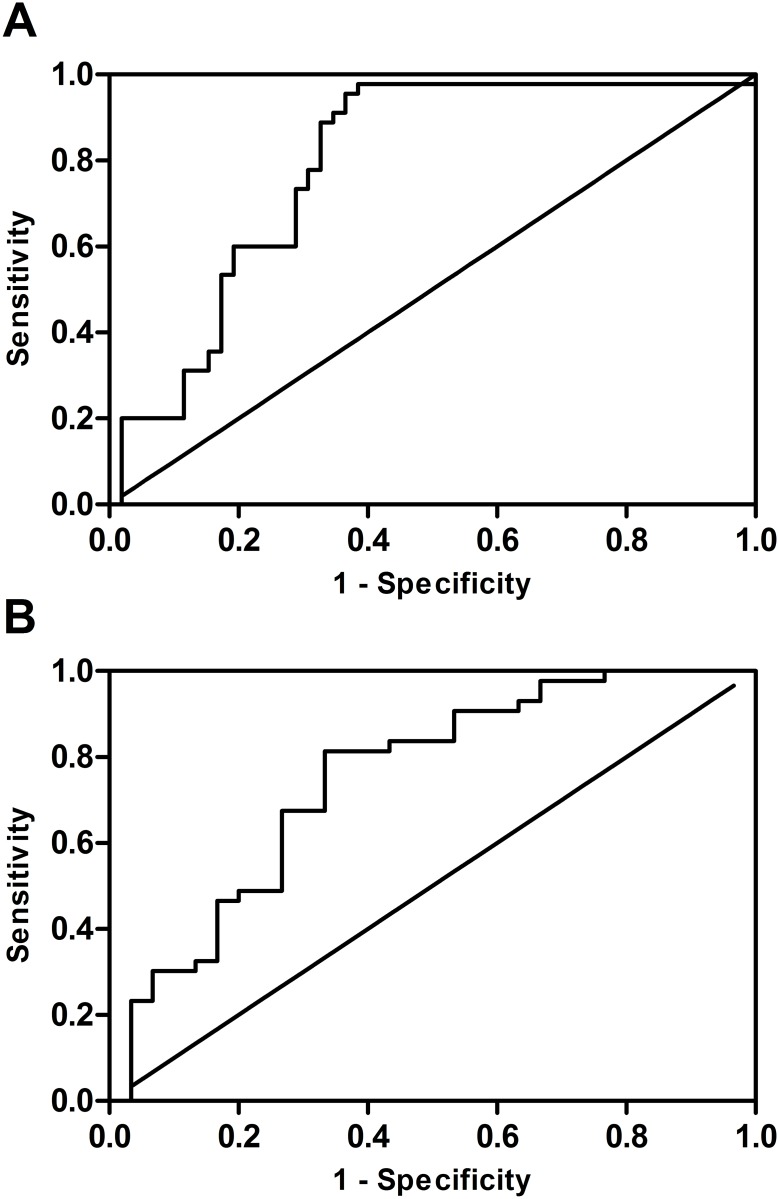
ROC curve analysis. Panel A: ROC curve for serum TFF2 and later CKD stages, AUC 0.79 (0.70–0.88, p < 0.001). Panel B: ROC curve for urine TFF2 and early CKD stages, AUC 0.75 (0.63–0.87, p < 0.001).

For ROC analysis data from patients in the early and the later CKD group were included (n = 33 and 46, respectively). As depicted in Fig 3, ROC curve analysis displayed an area under the curve for serum TFF2 of 0.79 (0.70–0.88, p < 0.001; Fig 3A) and one optimum cut-off value at 2.986 for the diagnosis of later CKD stages. For the differentiation of early vs. later CKD stages by TFF2 urine concentration, ROC curve analysis revealed an area under the curve of 0.75 (0.63–0.87, p < 0.001; Fig 3B) and one best cut-off value at 122.391.

## Discussion

We were able to demonstrate significantly higher TFF2 serum levels in patients with mid and later CKD stages as compared to healthy controls. Additionally, early CKD stages differed significantly from later stages. In contrast, TFF2 urine levels declined during CKD progression with significant lower levels in later CKD stages as compared to early or mid stages. TFF2 urine levels in early stages tended to be higher compared to controls, but did not reach statistical significance under the Bonferroni adjustment for multiple comparison. However, fractional TFF2 excretion was significantly higher in early CKD stages as compared to healthy probands. Significantly, the ROC curve analysis showed that TFF2 urine and serum levels may predict different CKD stages. Furthermore, patients suffering from vascular or diabetic nephropathy had significantly higher TFF2 serum levels as compared to patients with glomerulonephritis.

During the progression from kidney disease to end-stage renal failure, persistent inflammation triggers cell damage and tissue degeneration [5,6], often resulting in the need of kidney replacement therapy. To contain the progression of renal dysfunction, counter-regulations like the initiation of a heat-shock response are mounted to prevent cell damage and limit renal cell death [17,18]. TFF peptides hold important functions during epithelial protection and restitution. They facilitate epithelial cell migration to cover damaged areas, constrain proapoptotic signals and promote angiogenesis and leukocyte migration. TFF peptipes have been mostly studied in the gastrointestinal tract and its associated diseases of chronic inflammation such as inflammatory bowel disease, where upregulation of TFF peptides has been demonstrated [16,19].

Lately multiple studies have emphasized the importance of TFF peptipes also in other systems and associated diseases, such as the kidney and the urinary tract [9,10,15,20,21]. In patients with renal cell carcinoma expression of TFF peptides are highly upregulated along the urinary tract with TFF3 being the most prominent one [20]. Analyzed specimens were taken from non-pathological regions and signs of inflammation or neoplastic changes were exclusion criteria. However, the influence of carcinoma diseases on renal peptide expression cannot be finally excluded in non-pathological regions. The same study described increased urinary TFF2 levels in patients suffering from nephrolithiasis and associated TFF2 upregulation during urinary tract infection by so far unpublished results [20]. In patients with CKD increased levels of TFF1 and TFF3 in urine and serum were found and their expression correlated with renal function [9,10]. Consequently, also the involvement of TFF2 in CKD is very likely. To the best of our knowledge, this is the first study evaluating TFF2 levels in chronic renal injury.

Indeed, we were able to demonstrate higher TFF2 urine levels during early kidney diseases and that normalization of TFF2 serum levels is facilitated by increased fractional excretion. Over time, however, as kidney function further declines, the compensatory increase of TFF2 excretion is exhausted, which in turn leads to a successive increase of serum TFF2 levels. A non-significant trend towards increased urinary TFF2 levels in early CKD stages compared to controls was noticed and may reflect the described initial repair response and immediate clearance. In the gastrointestinal tract higher TFF peptide levels are associated with ulceration and inflammation and can be correlated with disease activity [16,19]. Similarly, TFF2 upregulation in chronic renal failure might occur to limit cell death and epithelial damage. The main production site of urinary TFF2 seems to be the kidney, as fractional TFF2 excretion rates above 1 point towards active tubular excretion.

Recently increased urinary TFF1 levels have been detected with the onset of CKD, which normalized with disease progression to levels comparable to that of healthy controls [10]. In contrast TFF3 urine excretion was low in healthy controls and early CKD stages but increased during disease progression. Again, TFF1 and TFF3 serum concentrations only increased with later CKD stages. Interestingly, studies in TFF1 knock-out mice revealed a reduced expression of TFF2, indicating a genetic co-regulation of TFF1 and TFF2 [22]. Their findings are in line with our data collected from urine and serum samples of CKD patients, strengthening the assumption of a TFF1 and TFF2 co-regulation. During the initial phase of renal diseases, TFF1 secretion is increased obviously by elevated urinary levels when compared to healthy probands or later CKD stages [10]. Consequently, higher urinary TFF1 levels and the potentially co-regulated TFF2 might reflect the initial acute phase of renal diseases. In animal models TFF1 and TFF2, but not TFF3 have been shown to be upregulated during the acute phase of gastrointestinal diseases, while TFF3 reemerged during the restitution phase [23–25]. Comparably, TFF3 levels have been shown to constantly increase during CKD progression to end-stage renal failure [9, 10].

ROC curve analysis revealed that by measurement of urine or serum TFF2, different CKD stages could be estimated. Moreover, TFF2 concentrations significantly correlated with serum creatinine, which is the most common routine clinical biomarker used to assess renal function. Furthermore, the contrary elevation of TFF2 in serum and urine could indicate changes in renal function and might offer potential to examine CKD course and treatment progression, as TFF2 levels significantly correlated with eGFR. This in turn could help to guide the individually adjusted treatment plan.

Besides varying TFF2 levels in different CKD stages, we found significantly increased TFF2 serum levels in patients with vascular or diabetic nephropathy as compared to patients with glomerulonephritis. Even though patient number within renal diseases is low, this finding allows a first insight into differently regulated TFF2 expression rates within various CKD entities.

Though this study generated promising results, we are aware of some methodological limitations. Although the used ELISA kit was reliable in recovery, sensitivity, linearity, and coefficient of variations, it was not designed for use in clinical testing. Samples had to be diluted to obtain TFF2 concentrations within the standard curve, which might have obliterated potential changes in protein levels. By testing undiluted samples a significant change in TFF2 urine concentrations between healthy probands and early CKD stages might be detectable. This adds to the importance of further clinical testing of TFF2 as biomarker for the early detection of CKD. Our study was initially designed to evaluate TFF2 levels irrespective of the underlying kidney disease. As a consequence, the number of patients suffering from rare CKD entities is low and might conceal important findings in those diseases. Therefore, the inclusion of additional patients with different renal diseases and CKD stages would increase statistical power and might help to unravel the role of TFF2 in the acute phase of renal failure and during progression to end-stage renal disease. Furthermore, histological examinations could identify the source of TFF2 expression in the affected kidney which in turn could help to understand the regulation of repair mechanisms within kidney diseases.

## Conclusion

We provide evidence of a differential TFF2 concentration pattern in urine and serum of patients suffering from CKD. Whereas TFF2 urine levels abated in mid and later stages as compared to early stages, serum TFF2 concentrations increased progressively in later stages. The disparate TFF2 concentration profile in urin and serum indicates changes in renal function and might offer potential to identify different CKD stages. However, larger clinical studies and longitudinal surveys will be necessary to reveal the role of TFF2 as biomarker in CKD and during progression to end-stage renal disease in different renal diseases.

## References

[1] Levey AS, Coresh J, Balk E, Kausz AT, Levin A, Steffes MW, et al (2003) National Kidney Foundation practice guidelines for chronic kidney disease: evaluation, classification, and stratification. Ann Intern Med 139: 137–147. 12859163 10.7326/0003-4819-139-2-200307150-00013

[2] System USRD (2014) 2014 annual data report: An overview of the epidemiology of kidney disease in the United States. National Institutes of Health, National Institute of Diabetes and Digestive and Kidney Diaseases, Bethesda, MD.

[3] Zoccali C (2006) Traditional and emerging cardiovascular and renal risk factors: an epidemiologic perspective. Kidney Int 70: 26–33. 10.1038/sj.ki.5000417 16723985

[4] Kinchen KS, Sadler J, Fink N, Brookmeyer R, Klag MJ, Levey AS, et al (2002) The timing of specialist evaluation in chronic kidney disease and mortality. Ann Intern Med 137: 479–486. 12230348 10.7326/0003-4819-137-6-200209170-00007

[5] Anders HJ, Vielhauer V, Schlondorff D (2003) Chemokines and chemokine receptors are involved in the resolution or progression of renal disease. Kidney Int 63: 401–415. 10.1046/j.1523-1755.2003.00750.x 12631106

[6] Lebherz-Eichinger D, Klaus DA, Reiter T, Horl WH, Haas M, Ankersmit HJ, et al (2014) Increased chemokine excretion in patients suffering from chronic kidney disease. Transl Res. 10.1016/j.trsl.2014.07.004 25168017

[7] Roth GA, Lebherz-Eichinger D, Ankersmit HJ, Hacker S, Hetz H, Vukovich T, et al (2011) Increased total cytokeratin-18 serum and urine levels in chronic kidney disease. Clin Chim Acta 412: 713–717. 10.1016/j.cca.2010.12.030 21195700

[8] Cook GA, Familari M, Thim L, Giraud AS (1999) The trefoil peptides TFF2 and TFF3 are expressed in rat lymphoid tissues and participate in the immune response. FEBS Lett 456: 155–159. 10452549 10.1016/s0014-5793(99)00940-0

[9] Du TY, Luo HM, Qin HC, Wang F, Wang Q, Xiang Y, et al (2013) Circulating serum trefoil factor 3 (TFF3) is dramatically increased in chronic kidney disease. PLoS One 8: e80271 10.1371/journal.pone.0080271 24282531 PMC3840008

[10] Lebherz-Eichinger D, Tudor B, Ankersmit HJ, Reiter T, Haas M, Roth-Walter F, et al (2015) Trefoil Factor 1 Excretion Is Increased in Early Stages of Chronic Kidney Disease. PLoS One 10: e0138312 10.1371/journal.pone.0138312 26390128 PMC4577124

[11] Kjellev S (2009) The trefoil factor family—small peptides with multiple functionalities. Cell Mol Life Sci 66: 1350–1369. 10.1007/s00018-008-8646-5 19099184 PMC11131466

[12] Otto WR, Thim L (2005) Trefoil factor family-interacting proteins. Cell Mol Life Sci 62: 2939–2946. 10.1007/s00018-005-5482-8 16374582 PMC11139177

[13] Taupin D, Podolsky DK (2003) Trefoil factors: initiators of mucosal healing. Nat Rev Mol Cell Biol 4: 721–732. 10.1038/nrm1203 14506475

[14] Coons SJ (2009) The FDA's critical path initiative: a brief introduction. Clin Ther 31: 2572–2573. 10.1016/j.clinthera.2009.11.035 20110002

[15] Astor BC, Kottgen A, Hwang SJ, Bhavsar N, Fox CS, Coresh J (2011) Trefoil factor 3 predicts incident chronic kidney disease: a case-control study nested within the Atherosclerosis Risk in Communities (ARIC) study. Am J Nephrol 34: 291–297. 10.1159/000330699 21829008 PMC3169359

[16] Vestergaard EM, Brynskov J, Ejskjaer K, Clausen JT, Thim L, Nexo E, et al (2004) Immunoassays of human trefoil factors 1 and 2: measured on serum from patients with inflammatory bowel disease. Scand J Clin Lab Invest 64: 146–156. 15115253 10.1080/00365510410001176

[17] Lebherz-Eichinger D, Ankersmit HJ, Hacker S, Hetz H, Kimberger O, Schmidt EM, et al (2012) HSP27 and HSP70 serum and urine levels in patients suffering from chronic kidney disease. Clin Chim Acta 413: 282–286. 10.1016/j.cca.2011.10.010 22032827

[18] Musial K, Zwolinska D (2013) Extracellular Hsp27 in patients with chronic kidney disease. Kidney Int 83: 971. 10.1038/ki.2013.33 23633061

[19] Gronbaek H, Vestergaard EM, Hey H, Nielsen JN, Nexo E (2006) Serum trefoil factors in patients with inflammatory bowel disease. Digestion 74: 33–39. 10.1159/000096591 17068395

[20] Rinnert M, Hinz M, Buhtz P, Reiher F, Lessel W, Hoffmann W (2010) Synthesis and localization of trefoil factor family (TFF) peptides in the human urinary tract and TFF2 excretion into the urine. Cell Tissue Res 339: 639–647. 10.1007/s00441-009-0913-8 20063012

[21] Chutipongtanate S, Nakagawa Y, Sritippayawan S, Pittayamateekul J, Parichatikanond P, Westley BR, et al (2005) Identification of human urinary trefoil factor 1 as a novel calcium oxalate crystal growth inhibitor. J Clin Invest 115: 3613–3622. 10.1172/JCI25342 16308573 PMC1288833

[22] Lefebvre O, Chenard MP, Masson R, Linares J, Dierich A, LeMeur M, et al (1996) Gastric mucosa abnormalities and tumorigenesis in mice lacking the pS2 trefoil protein. Science 274: 259–262. 8824193 10.1126/science.274.5285.259

[23] Itoh H, Tomita M, Uchino H, Kobayashi T, Kataoka H, Sekiya R, et al (1996) cDNA cloning of rat pS2 peptide and expression of trefoil peptides in acetic acid-induced colitis. Biochem J 318 (Pt 3): 939–944. 8836141 10.1042/bj3180939 PMC1217708

[24] Tran CP, Cook GA, Yeomans ND, Thim L, Giraud AS (1999) Trefoil peptide TFF2 (spasmolytic polypeptide) potently accelerates healing and reduces inflammation in a rat model of colitis. Gut 44: 636–642. 10205199 10.1136/gut.44.5.636 PMC1727500

[25] Xian CJ, Howarth GS, Mardell CE, Cool JC, Familari M, Read LC, et al (1999) Temporal changes in TFF3 expression and jejunal morphology during methotrexate-induced damage and repair. Am J Physiol 277: G785–795. 10516144 10.1152/ajpgi.1999.277.4.G785

